# Impaired Lymphocyte Profile in Schistosomiasis Patients with Periportal Fibrosis

**DOI:** 10.1155/2013/710647

**Published:** 2013-11-18

**Authors:** Luciana Santos Cardoso, Andréia de Souza Rocha Barreto, Jamille Souza Fernandes, Ricardo Riccio Oliveira, Robson da Paixão de Souza, Edgar M. Carvalho, Maria Ilma Araujo

**Affiliations:** ^1^Serviço de Imunologia, Hospital Universitário Professor Edgard Santos, Universidade Federal da Bahia (UFBA), 4110-160 Salvador-BA, Brazil; ^2^Departamento de Análises Clínicas e Toxicológicas, Faculdade de Farmácia, UFBA, 40171-115 Salvador-BA, Brazil; ^3^Instituto Nacional de Ciência e Tecnologia de Doenças Tropicais (INCT-DT/CNPq-MCT), Brazil; ^4^Centro de Pesquisa Gonçalo Moniz, FIOCRUZ, 40296-710 Salvador-BA, Brazil; ^5^Escola Bahiana de Medicina e Saúde Pública, 4050-420 Salvador-BA, Brazil

## Abstract

The Th2 immune response in chronic schistosomiasis is associated with the development of periportal fibrosis. However, little is known about the phenotype and activation status of T cells in the process. *Objective*. To evaluate the profile of T cells in schistosomiasis patients with periportal fibrosis. *Methods*. It was a cross-sectional study, conducted in the village of Agua Preta, Bahia, Brazil, which included 37 subjects with periportal fibrosis determined by ultrasound. Peripheral blood mononuclear cells were obtained by the Ficcol-hypaque gradient and the frequency of T cells expressing the surface markers CD28, CD69, CD25, and CTLA-4 was determined by flow cytometry. *Results*. The frequency of CD4^+^CD28^+^ T lymphocytes was higher in individuals with moderate to severe fibrosis compared to patients with incipient fibrosis. We did not observe any significant difference in the frequency of CD4^+^ T cells expressing CD69 among groups of individuals. There was also no significant difference in the frequency of CD8^+^ T cells expressing CD28 or CD69 among the studied groups. Individuals with moderate to severe fibrosis presented a lower frequency of CD8^+^ T cells, CD4^+^CD25^high^ T cells, and CD4^+^CTLA-4^+^ T cells when compared to patients without fibrosis or incipient fibrosis. The frequency of CD4^+^CD25^low^ cells did not differ between groups. *Conclusion*. The high frequency of activated T cells coinciding with a low frequency of putative Treg cells may account for the development of periportal fibrosis in human schistosomiasis.

## 1. Introduction

Schistosomiasis is a parasitic disease which accounts for the second place in terms of socioeconomic and public health burden in tropical and subtropical areas. It is a chronic and debilitating disease caused by parasites of the genus *Schistosoma. *About 200 million people are affected worldwide, and close to 800 million are at the risk of infection [1]. Liver pathology results from the host immune response to antigens from the eggs that become trapped in the portal venous system. The granulomas formed around the eggs act as barriers which prevent the dispersion of egg antigens of *S. mansoni*. However, about 5% of infected individuals evolve to periportal fibrosis which is associated with the morbidity and mortality described in chronic schistosomiasis [2–4].

Studies have evaluated the cytokine response associated with granuloma formation and development of periportal fibrosis due to schistosomiasis, both in experimental models and *in vitro* models of granuloma or tissue biopsies [5–7]. There are, however, few studies evaluating the lymphocyte profile in patients with periportal fibrosis and the participation of costimulatory molecules in the process. The activation process of T cells requires stimulation through costimulatory molecules which interact with corresponding ligands on antigen presenting cells (APC). One of the most important costimulatory molecules on T cells is CD28, which is constitutively expressed and binds to its ligands on APCs, CD80, and CD86. CD86 is constitutively expressed at low levels, and it is rapidly upregulated after primary antigen recognition, whereas CD80 exhibits delayed expression kinetics [8]. Another ligand for CD80 and CD86 is CTLA4. The expression of this molecule is rapidly upregulated after T cell activation and provides a negative feedback signal limiting the immune response [9]. In addition to costimulatory molecules expressed during the antigen presentation process, we also evaluated in this study the expression of CD69, a molecule associated with T cell activation [10]. This molecule is expressed following activation of T cells, and it induces proliferation of these cells through increased synthesis of IL-2 [10]. The CD4^+^ T cells, depending on the cytokine environment that is exposed at the time of antigen presentation, can differentiate into effector T cells subtypes of Th1, Th2, and Th17 or T regulatory (Treg) phenotypes. Treg cells have been described to play a key role in controlling the immune system. Depending on how they are derived, regulatory T cells can be classified into two main types. The natural regulatory cells arise from the thymus as a distinct subtype of mature T cell and express the alpha chain of the IL-2 receptor, the molecule CD25. They can also express the transcription factor, Foxp3 [11, 12]. Another type of regulatory cell, the regulatory T cells induced type 1 (Tr1), develops from the differentiation of naive T cells in the periphery after contact with high concentrations of antigen [13–15]. In recent years, the expression of CD25 on T lymphocytes has been better characterized. Responder or activated T cells are those which show low expression of CD25 (CD4^+^CD25^low^) or did not express this molecule (CD4^+^CD25^neg^). Cells expressing high levels of CD25 (CD4^+^CD25^high^), on the other hand, have potent suppressor activities on the immune response, by inhibiting cell proliferation and cytokine secretion by T cells. These cells can act via cell-cell contact [16, 17] or by the synthesis of regulatory cytokines, such as IL-10 and TGF-*β* [18].

We hypothesize that the process of periportal fibrosis development in schistosomiasis is associated with an exacerbated activation status of T cells due to an impairment of regulatory molecule expression in these cells. To test this hypothesis, we evaluated the phenotype and the degree of activation of T lymphocytes in patients with varying degrees of periportal fibrosis secondary to schistosomiasis. 

## 2. Patients, Materials and Methods

### 2.1. Study Design and the Endemic Area

This study was carried out in an endemic area of schistosomiasis named Água Preta, in the state of Bahia, Brazil. Água Preta is located 280 km south of Salvador, the capital of the state of Bahia. It is composed of a residential area in the center of the village and some surrounding farms. A total of approximately 800 people live in the community. They live in poor sanitary conditions, and agriculture is the predominant occupation. There is one river in this region that is used for bathing, washing clothes and utensils, and leisure—exposing the residents to high risk of *Schistosoma* infection.

A cross-sectional parasitological survey using Kato-Katz [19] and sedimentation techniques was conducted on three different stool samples collected on different days. The inclusion criteria for this study were individuals from endemic areas who have at least one positive parasitological exam for *S. mansoni*. From the 537 individuals who agreed to participate in this study 334 were infected with *S. mansoni* (62.5%). The frequency of other helminthic infections was 43.4% for *Trichuris trichiura*, 37.4% for* Ascaris lumbricoides*, 33.7% for hookworms, and 3.5% for *Strongyloides stercoralis*. From 334 individuals who were infected with *S. mansoni*, 220 agreed to perform abdominal ultrasound, in order to determine the degree of periportal fibrosis [20]. They also agreed to donate blood for the study of the immunological response. For this particular aim, we analyzed fifteen patients with grade 0 (without fibrosis), fifteen patients with grade I (incipient fibrosis), and seven with fibrosis of grades II and III (moderate and severe fibrosis). We had difficulty in finding patients with advanced stages of fibrosis in the region of the study; only seven patients with this condition met the inclusion criteria. We did not include individuals less than 05 or above 60 years old. We also did not include alcoholic individuals and those with positive serology for HIV, HTLV-1, or hepatitis virus type B and C—all of which are conditions that could interfere with the immunological response.

### 2.2. Ultrasound Examination

Abdominal ultrasounds (USG) were performed using the Quantum 2000 Siemens and Elegra Siemens ultrasound with a convex transductor of 3.5–5.0 MHz. Liver span was measured at the midclavicular line and midline. The liver was also examined for smoothness of surface; echogenicity and posterior attenuation of the sound beam; and portal vein diameter outside the liver midway between its entrance into the portal hepatic and its first bifurcation in the liver. Periportal fibrosis was observed as multiple diffuse echogenic areas. Grading of periportal fibrosis was determined by the mean total thickness of four portal tracts after the first division from the right and left branches of portal vein (PT1) as follow: degree 0, mean thickness: <3 mm; degree I, mean thickness: 3 to 5 mm; degree II, mean thickness: >5 to 7 mm; and degree III, mean thickness: >7 mm [21, 22]. Of the 220 individuals evaluated 62 (28.2%) had some degree of periportal fibrosis [20]. The scores of periportal fibrosis were grouped according to the severity, assigning degree 0 to those without periportal fibrosis, degree I to individuals with incipient periportal fibrosis, and degrees II and III to those with moderate to severe periportal fibrosis [23].

To perform the immune response, we included 15 individuals with degree 0 and 15 individuals with degree I, while all individuals with severe forms of the disease characterized by the grade II or III (*n* = 07) were included. 

### 2.3. Fecal Examinations for Parasite Load

Three stool samples from each individual were examined using the Hoffman sedimentation method, to identify helminths and enteric protozoa, and Kato-Katz method, to estimate the number of eggs per gram of feces [19].

### 2.4. *Ex Vivo* Staining

**Figure 1:**
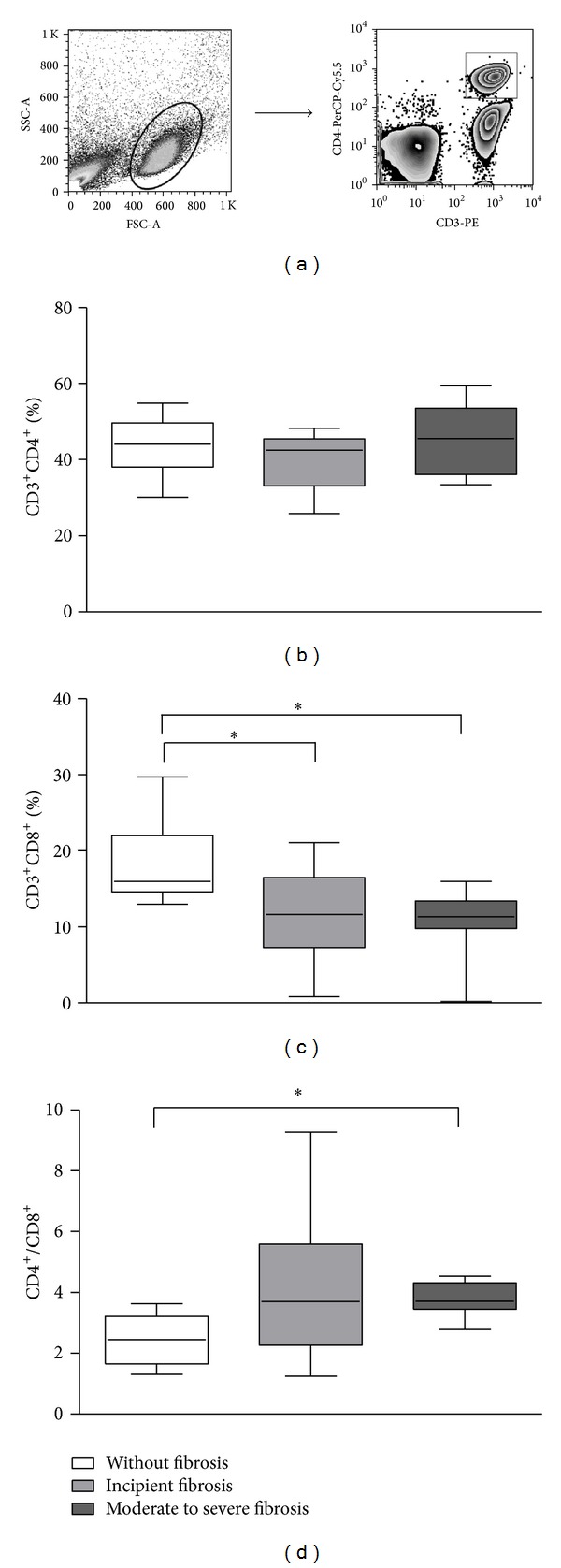
Strategy for T cell evaluation by flow cytometry. The cell populations were defined by nonspecific fluorescence from the forward (FSC) and side scatter (SSC) as parameters of cell size and granularity, respectively (a). Lymphocyte regions were determined through the expression of CD3-PE and CD4-PeCy7 (b) or CD3-PE and CD8-FITC (c). Frequency of TCD4^+^ cells and TCD8^+^ cells and ratio between TCD4^+^ and TCD8^+^ cells (d) in individuals infected with *S. mansoni* with different degrees of periportal fibrosis. Results are expressed in median, minimum, maximum, and interquartile values of frequency (*P < 0.05; Kruskal-Wallis test).

The PBMC were labeled with monoclonal antibodies for the surface markers, CD3, CD4, CD8, CD25, CD28, CD69, and CTLA-4, with the following fluorochromes FITC (anti-CD3, anti-CD4, and anti-CD25), PercPCy5.5 (anti-CD4, anti-CD28), PerCP (anti-CD69), APC (anti-CD4), PE (anti-CD3 and anti-CTLA-4), and PeCy7 (anti-CD8). The stained cells, 3 × 10^5^ cells/mL in 30 uL of a solution, were added to each well of 96 well plate. Then, 20 uL of diluted antibody solution was added. The plates were incubated, protected from the light for 20 minutes at 4°C. After staining, preparations were washed with 0.1% sodium azide PBS, fixed with 200 mL of 4% formaldehyde in PBS, and kept at 4°C for later acquisition using a flow cytometer (FACS canto: BD Biosciences). The cell populations were defined by the size parameters (FSC) and cell granularity (SSC) for the delimitation of the lymphocyte region. Limits for the quadrant markers were always set based on negative populations and control isotypes. Cells were analyzed according to the frequency of expression of cell surface markers using Flow Jo software (Tree Star, USA; Figure 1(a)).

### 2.5. Statistical Analysis

Statistical analyses were performed using the software Statistical Package for Social Science (version 9.0 for Windows; SPSS). The frequency of positive cells was expressed as percentages. The differences between means were assessed using an unpaired *t*-test to compare two groups or one-way ANOVA to compare three or more groups. Fisher's exact test was used to compare proportions. The difference in mean age was assessed by the Mann-Whitney test. Statistical significance was established at the 95% confidence interval.

## 3. Results

The demographic characteristics, parasite burden, and the ultrasonography evaluation of individuals included in this study are shown in the Table 1. The mean age of individuals with moderate to severe periportal fibrosis was higher than that in patients without fibrosis (*P* < 0.05). There was no significant difference in parasite burden among the groups of patients, and there was also no significant difference in gender distribution between groups (Table 1).

### 3.1. Frequency and Activation Status of CD4^+^ and CD8^+^ T Lymphocytes in Individuals with Different Degrees of Periportal Fibrosis due to *Schistosoma mansoni* Infection

While the frequency of CD3^+^CD4^+^ T cells was similar among patients with different degrees of periportal fibrosis (Figure 1(b)), a lower frequency of CD8^+^ T cells was observed in individuals with moderate to severe fibrosis (median = 11.4%; min–max = 0.2%–16%), compared to those without fibrosis (16%; 13%–29.7%) or with incipient fibrosis (11.6%; 0.8%–21.1%; *P* < 0.05; Figure 1(c)). Moreover, the CD4^+^/CD8^+^ T cell ratio was higher in individuals with moderate to severe fibrosis (3.7; 2.7–4.5) compared to those without fibrosis (2.4; 1.3–3.6; *P* < 0.05; Figure 1(d)). 

**Figure 2:**
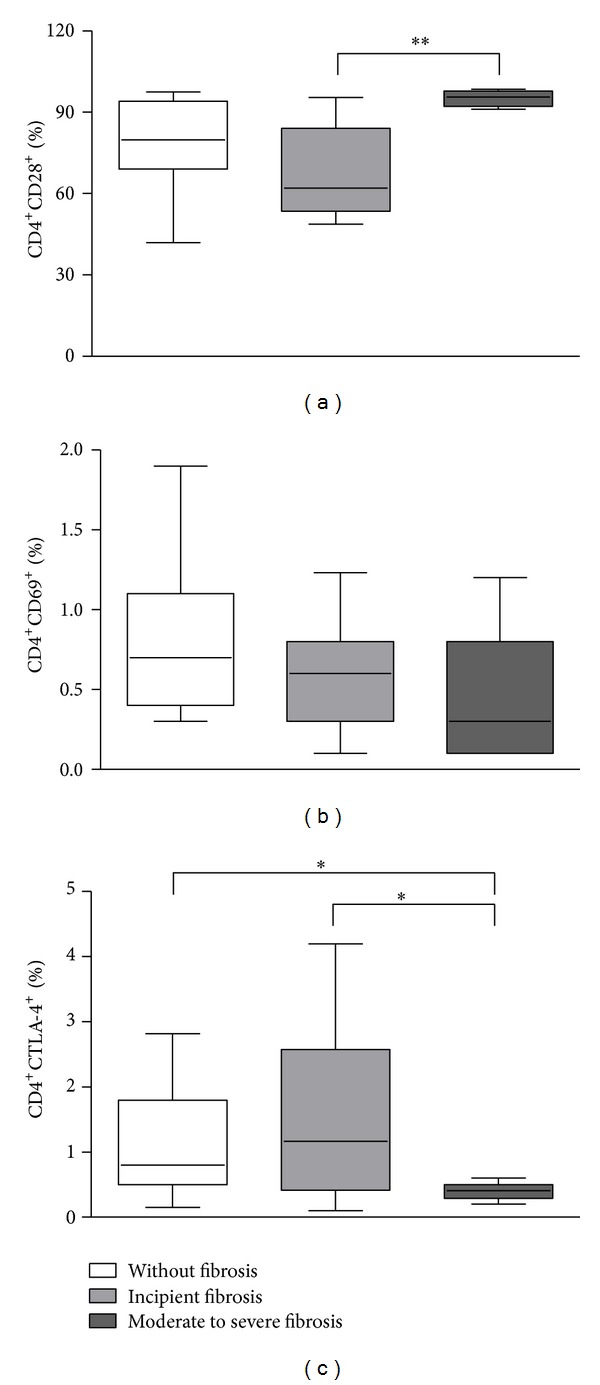
Frequency of T cell populations expressing costimulatory molecules. Frequency of T CD4^+^CD28^+^ (a), T CD4^+^CD69^+^ (b), and T CD4^+^CTLA-4^+^ cells (c) in individuals infected with *S. mansoni* with different degrees of periportal fibrosis. *Ex vivo* PBMC were stained for surface markers. The selection of T lymphocyte subsets was initially performed based on their forward (FSC) and side (SSC) light scatter properties, followed by immunophenotyping using a double-labeling platform with anti-CD4-FITC, anti-CD28-PE, anti-CD69-PerCP, and anti-CD152-PeCy5 mAbs and flow cytometry. Results are expressed in median, minimum, maximum, and interquartile values of frequency (*P < 0.05, **P < 0.005; Kruskal-Wallis test).

In this study, we also evaluated the frequency of T cells expressing some molecules associated with T cell activation status, such as CD28, CD69, and CTLA-4. The frequency of CD4^+^CD28^+^ T lymphocytes was higher in individuals with moderate to severe fibrosis (95.7%; 91.2%–98.5%) compared to patients with incipient fibrosis (62%; 48.8%–95.4%; *P* < 0.05; Figure 2(a)). We did not observe any significant difference in the frequency of CD4^+^ T cells expressing CD69 among groups of individuals (Figure 2(b)). There was also no significant difference in the frequency of CD8^+^ T cells expressing CD28 or CD69 among the studied groups (not shown).

Regarding the expression of CTLA-4, individuals with moderate to severe fibrosis presented lower frequency of CD4^+^CTLA-4^+^ T cells (0.41%; 0.2%–0.6%), compared to individuals without fibrosis (0.8%; 0.15%–2.8%) or with incipient fibrosis (0.16%; 0.1%–4.2%; *P* < 0.05; Figure 2(c)).

### 3.2. Subpopulations of CD4^+^CD25^+^ T Cells on Patients with Periportal Fibrosis due to *S. mansoni* Infection

**Figure 3:**
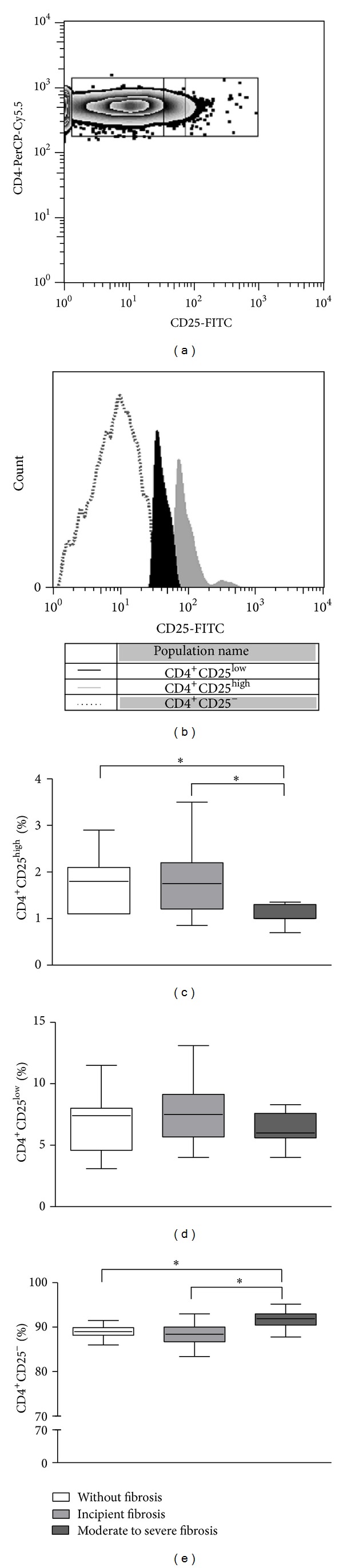
Frequency of T cell populations expressing CD25 molecule. Strategy to analyze the intensity of expression of CD25 molecule on TCD4^+^ cells. Plot (a) and histogram (b) are representative of one experiment. Frequency of TCD4^high^ (c), TCD4^low^ (d), and TCD4^neg^ cells (e) in individuals infected with *S. mansoni* with different degrees of periportal fibrosis. *Ex vivo* PBMC were stained for surface markers. The selection of T lymphocyte subsets was initially performed based on their forward (FSC) and side (SSC) light scatter properties, followed by immunophenotyping using a double-labeling platform with anti-CD4-APC and anti-CD25-FITC mAbs and flow cytometry. Results are expressed in median, minimum, maximum, and interquartile values of frequency (*P < 0.05; Kruskal-Wallis test).

It has been demonstrated that within the CD4^+^CD25^+^ T cell populations, those which express high levels of CD25 exhibit regulatory functions [16]. The intensity of CD25 expression in T cells was evaluated in this study (Figures 3(a) and 3(b)). The frequency of CD4^+^CD25^high^ T cells was lower in individuals with moderate to severe fibrosis (1.0%; 0.7%–1.35%) compared to individuals without fibrosis (1.8%; 1.1%–2.9%) or with incipient fibrosis (1.75%; 0.85%–3.5%; *P* < 0.05; Figure 3(c)).

The frequency of CD4^+^CD25^low^ T cells (Figure 3(d)) did not differ among groups, being 7.4% (3.1%–11.5%) in the group of individuals without fibrosis, 7.5% (4%–13.1%) in the group with incipient fibrosis, and 6% (4%–8.3%) in the group of individuals with moderate to severe fibrosis (*P* > 0.05). Individuals with moderate to severe fibrosis had, however, high frequency of TCD4^+^CD25^neg^ T cells (91.9%; 87.8%–95.2%) compared to those without fibrosis (89%; 86%–91.5%) or with incipient fibrosis (88.4%; 83.4%–93%; *P* < 0.05; Figure 3(e)).

## 4. Discussion

In this study, we evaluated the frequency of T lymphocyte subtypes and activation status of these cells in individuals with different degrees of periportal fibrosis due to *S. mansoni* infection. Most of the included subjects were female; however there was no difference in gender distribution between groups of patients with different degrees of fibrosis. The parasite load of *S. mansoni* was also similar between groups. Patients with moderate to severe fibrosis were older than individuals without periportal fibrosis, which corroborates with Oliveira and colleagues [23], who observed that the development of periportal fibrosis is a slow process usually occurring after 50 years of age.

Regarding the immune response, the frequency of CD4^+^ T cells did not differ between groups of patients with different degrees of periportal fibrosis, while CD8^+^ T cells were more frequent in individuals with moderate to severe fibrosis. Granuloma formation is mainly mediated by CD4^+^ T cells specific for the parasite antigens [4, 24], and these cells also participate in the modulatory process of granuloma development [25]. Studies have shown that both CD4^+^ and CD8^+^ T cells are sources of IL-10, a cytokine with suppressive activity that is highly produced during chronic *S. mansoni* infection [26]. We did not find, however, differences in the frequency of CD4^+^ T cells between groups of patients with different degrees of fibrosis, indicating that even with similar frequency of these cells, their profile might be important, and this aspect was also evaluated in this study. Regarding the CD8^+^ lymphocytes, there is some evidence that they participate in the control of granuloma formation and prevent pathology during chronic schistosomiasis [27]. This is supported by the data that show that there is an increased population of activated CD8^+^ T cells in individuals with the intestinal form of the disease [28].

In relation to the activation status of T lymphocytes in this study, we observed higher frequency of CD4^+^ T cells expressing CD28 in subjects with moderate to severe fibrosis when compared to cells from individuals with incipient fibrosis. There was, however, no difference in the frequency of CD8^+^CD28^+^ T cells among the groups. These findings suggest that expression of CD28 on CD4^+^ T cells may be associated with the development of more severe forms of schistosomiasis. The molecule CD69 is other marker of T cell activation. It has not been found on resting cells, as it is rapidly expressed following T cells activation. The expression of CD69 induces proliferation of T cells through the increased synthesis of IL-2 [10]. In this study, we observed no differences in the frequency of CD4^+^CD69^+^ and CD8^+^CD69^+^ T cells among patients with different degrees of periportal fibrosis. A lower expression of CD69 on eosinophils has been shown in patients with chronic intestinal schistosomiasis when compared to those with periportal fibrosis [29]. There are, however, few studies in the literature that assess the function of the CD69 molecule expressed on T lymphocytes in individuals infected with *S. mansoni. *CD69 expression in these cells may not be as important as on eosinophils. Indeed, the expression of CD69 on eosinophils of other Th2 mediated diseases, such as asthma, is associated with airway inflammation and severity of the disease [30, 31].

Regarding the costimulatory molecule, CTLA-4, we observed a lower frequency of TCD4^+^CTLA-4^+^ T cells in patients with moderate to severe fibrosis than in individuals with incipient fibrosis or without fibrosis. This suggests the involvement of lymphocytes expressing CTLA-4 as a mechanism to control the inflammatory process associated to liver fibrosis during chronic schistosomiasis. In an experimental study of *S. mansoni* infection, it was demonstrated that cells expressing CTLA-4 can control morbidity through a decrease in the Th2 immune response and in the eosinophil number [32].

In this study, we also evaluated the expression of CD25 on lymphocytes of schistosomiasis patients. While there was a high frequency of CD4^+^CD25^neg^ T cells in subjects with moderate to severe fibrosis, the frequency of CD4^+^CD25^high^ T cells was lower, compared to patients without fibrosis or incipient fibrosis. This finding is in agreement with what was found by Teixeira-Carvalho and colleagues [26], who demonstrated for the first time a high frequency of CD4^+^CD25^high^ T cells in patients with intestinal form of schistosomiasis, suggesting that they are key cells in controlling morbidity during chronic schistosomiasis. Experimental evidence points to the possible involvement of regulatory T cells in other chronic helminth infections, such as those caused by hookworm, where there is an increased number of circulating CD4^+^CD25^+^Foxp3^+^ T cells, compared to healthy individuals [33]. 

## 5. Conclusions

The data of high frequency of T activated cells in patients with periportal fibrosis, together with a low frequency of cells expressing regulatory markers in these patients, reinforces the hypothesis that the lack of modulatory mechanisms is associated with pathology in human schistosomiasis.

## Figures and Tables

**Table 1:** Demographic characteristics of the individuals enrolled in the study.

	Without fibrosis (*n* = 15)	Incipient fibrosis (*n* = 15)	Moderate to severe fibrosis (*n* = 07)	*P*
Age (years)*				
Median (min–max)	30 (12–50)	34 (20–62)	47 (26–70)	<0.05^a^
Gender				
Male (%)**	06 (40)	05 (33.3)	02 (28.5)	>0.05
*S. mansoni* parasite load				
Median (min–max; eggs per gram of feces)	72 (24–392)	72 (24–600)	72 (24–192)	>0.05

^a^Without fibrosis versus moderate to severe fibrosis. *ANOVA; **Fisher's exact test.
